# Association between sagittal balance and adjacent segment degeneration in anterior cervical surgery: a systematic review and meta-analysis

**DOI:** 10.1186/s12891-019-2800-0

**Published:** 2019-09-14

**Authors:** Yijian Zhang, Yijie Shao, Hao Liu, Junxin Zhang, Fan He, Angela Chen, Huilin Yang, Bin Pi

**Affiliations:** 1 Department of Orthopedics, The First Affiliated Hospital of Soochow University, 899, Pinghai Road, Suzhou, 215006 China; 2 Orthopedic Institute, Soochow University, Suzhou, 215006 China; 3 Department of School of Public Health and Health Systems, Faculty of Applied Health Sciences Biochemistry Department, University of Waterloo, Waterloo, Canada

**Keywords:** Sagittal balance, Adjacent segment degeneration, Cervical degenerative disease, Anterior cervical surgery

## Abstract

**Background:**

ASD is a relatively common degenerative alteration after cervical surgery which occurs above or below the fused segment. In addition, some patients may need reoperation to treat severe ASD after the primary surgery. It was considered that sagittal balance is correlated with postoperative clinical outcomes; however, few studies have reported the influence of sagittal balance on ASD. The present study is designed to investigate whether sagittal balance impacts the pathology of adjacent segment disease (ASD) in patients who undergo anterior cervical surgery for degenerative cervical disease.

**Methods:**

Databases including Pubmed, Embase, Cochrane library, and Web of Science were used to search for literature published before June 2018. Review Manager 5.3 was used to perform the statistical analysis. Sagittal balance parameters before and after surgery were compared between patients with and without ASD. Weighted mean difference (WMD) was summarized for continuous data and *P* < 0.05 was set for the level of significance.

**Results:**

A total of 221 patients with ASD and 680 patients without ASD from seven articles were studied in this meta-analysis. There were no significant differences in most sagittal balance parameters between the two groups, except for postoperative cervical lordosis (CL) (WMD -3.30, CI -5.91, − 0.69, *P* = 0.01).

**Conclusions:**

Some sagittal balance parameters may be associated with the development of ASD after anterior cervical surgery. Sufficient restoration of CL may decrease the incidence of ASD. The results in present study needed to be expanded carefully and further high-quality studies are warranted to investigate the impact of sagittal balance on ASD.

**Electronic supplementary material:**

The online version of this article (10.1186/s12891-019-2800-0) contains supplementary material, which is available to authorized users.

## Introduction

Cervical degenerative disease is common among elderly people, and manifests as neck pain, cervical radiolopathy and myelopathy [1]. Clinical symptoms, caused by compression of the nerve root and spinal cord, may include disc protrusion, spondylotic bone spurs, and ligament thickening [2]. The surgical procedure for treating this disease can effectively improve radiographic and clinical outcomes, and has been frequently used in the past few decades [3]. Compared to other approaches, anterior procedures including anterior cervical discectomy and fusion (ACDF), anterior cervical corpectomy and fusion (ACCF), cervical disc arthroplasty (CDA), and total disc replacement (TDR) can restore cervical lordosis better and achieve preferred exposure and decompression [4, 5]. However, anterior cervical surgery has many drawbacks such as cage subsidence, dysphagia, and elevated rates of pseudoarthrosis [6].

Adjacent segment disease (ASD) refers to recurrent radicular or myelopathic symptoms from adjacent degeneration after surgery [7]. It has been recognized as an important complication after anterior cervical discectomy and fusion, and may be related to many factors [7]. A meta-analysis by Wang et al. [8] reported a 6.2% incidence of ASD after single-level ACDF and found that old age and the development of canal stenosis were risk factors for ASD. Bydon et al. [9] analyzed the location and length of arthrodesis for index ACDFs and found that stress and instability introduced during surgery may result in the pathogenesis of ASD. Unfortunately, the exact etiology of ASD has not yet been illuminated.

Recently, the restoration of sagittal balance after cervical surgery has been emphasized more frequently. Postoperative sagittal balance of the cervical spine is considered to be correlated with clinical outcomes. Kato et al. [10] reported an association between postoperative cervical deformity and outcomes which indicate that poor cervical alignment after surgery may predict a worse SF-36 PCS. Furthermore, previous studies revealed that sagittal balance was related to postoperative ASD. Faldini et al. [11] found that a postoperative segmental alignment greater than 0° increased the incidence of adjacent-level degeneration. Hu et al. [12] also reported that patients without improved cervical lordosis had a higher incidence of ASD, worse NDI scores and neck pain. Conversely, some studies reported no impact of sagittal balance on the progression of ASD [13]. Hence, we aim to investigate whether sagittal balance of the cervical spine influences postoperative ASD after anterior cervical surgery by reviewing the previous literature.

## Methods

### Search strategy

This systematic review and meta-analysis followed the according PRISMA statement and guidelines [14, 15]. The literature research was performed using Pubmed, Embase, Cochrane library, and the Web of Science before June 2018. The search terms combined the following items: “sagittal balance”, “sagittal alignment”, “sagittal imbalance”, “cervical lordosis”, “segmental lordosis”, “sagittal vertical axis”, “T1 slope”, “adjacent segment disease”, and “adjacent segment degeneration”. The reference lists of all articles were reviewed for additional information and the language was restricted to English. The identified articles were evaluated independently by two reviewers (B P and YJ Z) in terms of the inclusion criteria.

### Selection criteria

The eligible studies included in this systematic review and meta-analysis met the following inclusion criteria: (1) randomized controlled trials or comparative studies (retrospective and prospective) of patients undergoing anterior cervical surgery; (2) patients were divided into two groups: those with ASD and those without ASD; (3) measured at least one sagittal balance parameter of the cervical spine; (4) follow-up time more than 1 year. The studies were excluded if: (1) sagittal balance parameters were not measured; (2) patients were not grouped by the presence of ASD; (3) abstracts, case reports, conference presentations, editorials and expert opinions; (4) biomechanical or corpse research. The Newcastle-Ottawa Scale was applied for evaluating of the quality of included studies.

### Data extraction

All potential studies were extracted from full texts, tables, and figures and assessed by two reviewers (B P and YJ Z) independently for inclusion. Discrepancies and doubtful cases were discussed to reach consensuses. The authors, year of publication, type of study, patient characteristics, surgical procedures, follow-up time, and sagittal balance parameters of the cervical spine (cervical lordosis, segmental lordosis, cervical range of motion, segmental range of motion) before and after surgery were all extracted from eligible studies.

### Statistical analysis

The statistical analysis was conducted using Review Manager Version 5.3 (Nordic Cochrane Centre, Cochrane Collaboration, Copenhagen, Sweden). The mean and standard deviation were integrated into the weighted mean difference (WMD) for continuous parameters, and a 95% confidence interval (CI) was used for statistical analysis. *P* values less than 0.05 were considered statistically significant. Both fixed- and random- effect models were tested. *x*^2^ tests and *I*^2^ statistics were used to study the heterogeneity between trials. The random-effects model was used when heterogeneity was significant (*I*^2^ > 50% and *P* < 0.10); otherwise, the fixed-effects model was used.

## Results

### Included studies

**Fig. 1:**
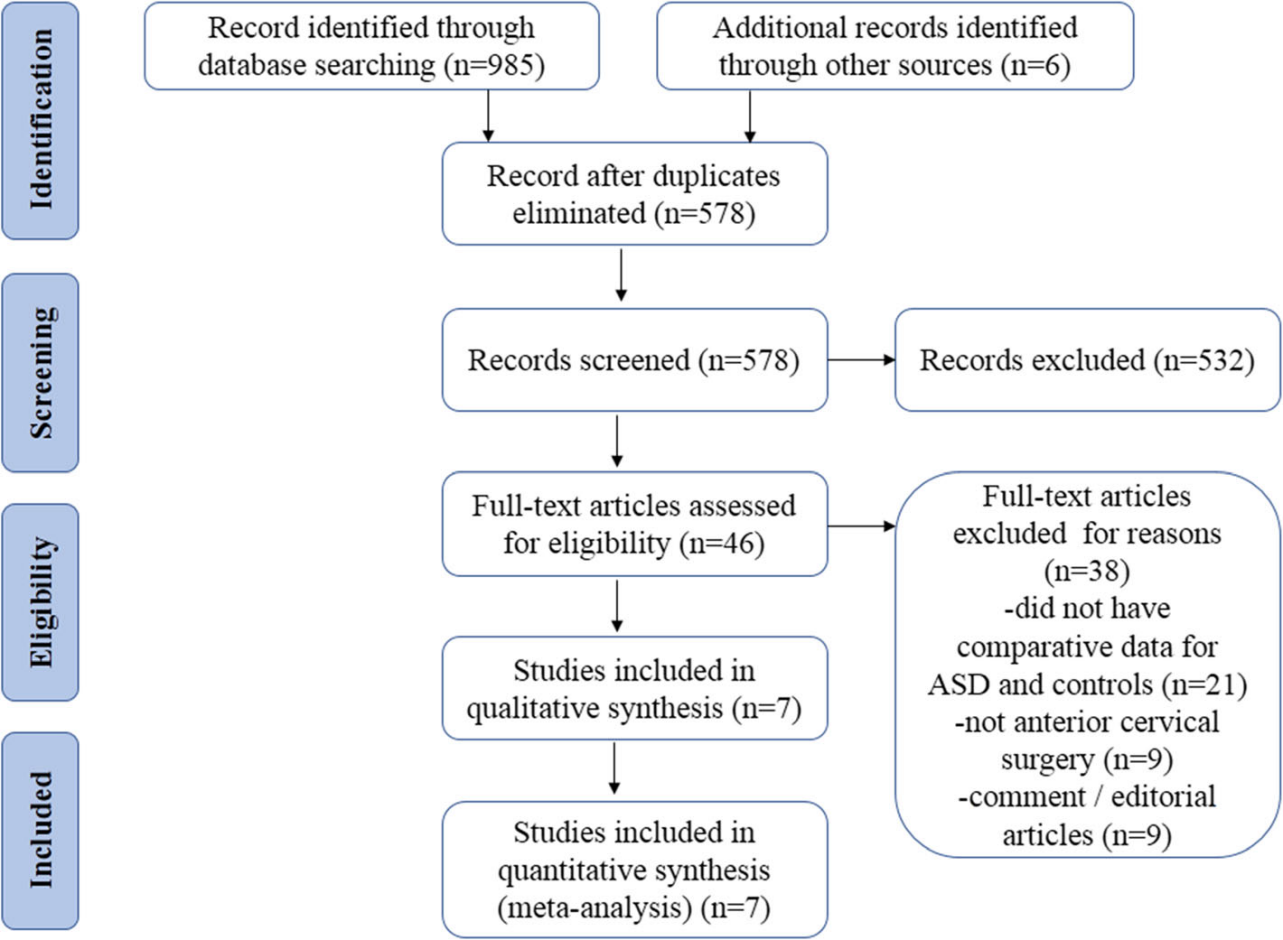
Flowchart of the selection of literatures

A flow diagram outlining the systematic review process is shown in Fig. 1. A total of 991 studies were found (985 searches identified through database and 6 identified through other sources). After elimination of duplicates, 578 records were screened, and 46 full-text articles were assessed for eligibility. Thirty-nine articles were subsequently excluded for having no comparative data, no anterior cervical surgery, or being comment/editorial articles. Ultimately, a total of seven articles were included in this meta-analysis (Table 1). Sagittal balance parameters measured in all studies included preoperative cervical lordosis (CL) (*N* = 4), segmental lordosis (SL) (*N* = 5), cervical range of motion (*N* = 5), segmental range of motion (*N* = 5), postoperative cervical lordosis (CL) (*N* = 4), segmental lordosis (SL) (*N* = 3), cervical range of motion (*N* = 4), and segmental range of motion (*N* = 4). All the included studies attained favorable Newcastle-Ottawa Scale (scores larger than 7 points), indicating a reliability of these studies (Table 2).

**Table 1:** Characteristics of included studies

Authors	Year	Study type	Age (years)	Male	Surgical procedure	Follow-up	N (ASD)	N (Controls)
Katsuura et al. [16]	2001	Retrospective study	48.1 for ASD and 52.7 for Control	19/21 for ASD and 15/21 for Control	ACDF	122.4 months for ASD and 112.7 months for Control	21	21
Kim et al. [17]	2015	Prospective study	43.52	117/180	TDR	Mean 48.6 months	73	107
Lee et al. [18]	2015	Retrospective study	47.1	12/14	CDA	Mean 43.4 months	5	9
		Retrospective study	53.6	24/28	ACDF	Mean 44.6 months	16	12
Li et al. [19]	2015	Retrospective study	50.2	61/106	ACDF	30.6 months for ASD and 31.2 months for non-ASD	28	88
Song et al. [20]	2018	Retrospective study	51.8 for ASD and 48.5 for N-ASD	11/25 for ASD and 85/175 for N-ASD	ACCF and ACDF	34.2 months for ASD and 34.8 months for N-ASD	25	175
Song et al. [21]	2014	Retrospective study	57.73 for ASD and 54.09 for Control	12/15 for ASD and 125/216 for Control	ACDF	Mean 63.85 months	15	216
Yang et al. [22]	2017	Retrospective study	43.3 for ASD and 42.6 for N-ASD	17/21 for ASD and 25/27 for N-ASD	CDA	Mean 79.3 months for SASD and 78.6 months Non-SASD	38	52

*Abbreviation*: *ASD* adjacent segment disease, *ACDF* anterior cervical discectomy and fusion, *CDA* cervical disc arthroplasty

**Table 2:** Quality assessment of the enrolled studies

Authors	Selection	Comparability	Exposure
Katsuura2001 et al [35]	★★★★	★★	★★★
Kim2015 et al [36]	★★★★	★★	★★★
Lee2015 et al [37]	★★★★	★★	★★★
Li2014 et al [38]	★★★★	★★	★★
Song2014 et al [39]	★★★	★★	★★★
Song2018 et al [40]	★★★★	★★	★★
Yang2017 et al [41]	★★★	★★	★★★

### Patients cohort

ASD was defined as the presence of at least one of the following criteria in radiographic finding: (1) new or enlarging anterior or posterior osteophyte formation; (2) new or developed calcification of the anterior or posterior longitudinal ligament; (3) new occurrence of a disc space narrowing less than 30% of the intervertebral disc space. A total of 221 patients with ASD and 680 patients without ASD were included in this meta-analysis. The mean age was 48.7 years in the ASD group (*N* = 4) and 50.6 in the non-ASD group (*N* = 4), which created no significant difference (*P* = 0.36). In addition, there was no significant difference in the proportion of males between the ASD (59/99, *N* = 4) and non-ASD groups (250/464, *N* = 4) (*P* = 0.40).

### Sagittal balance parameters

**Fig. 2:**
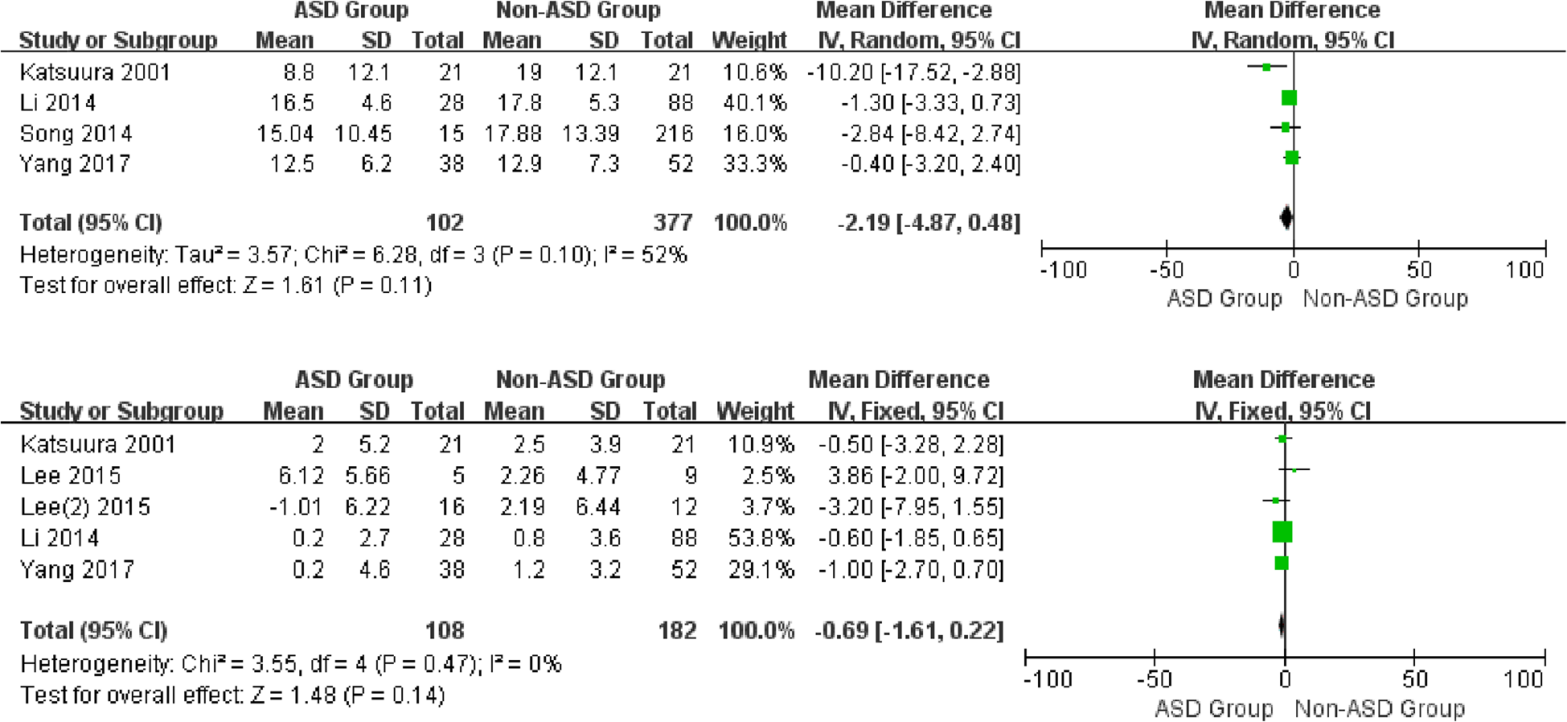
Forest plot of ASD group and non-ASD group: Comparison of preoperative cervical lordosis (above) and preoperative segmental lordosis (below)

**Fig. 3:**
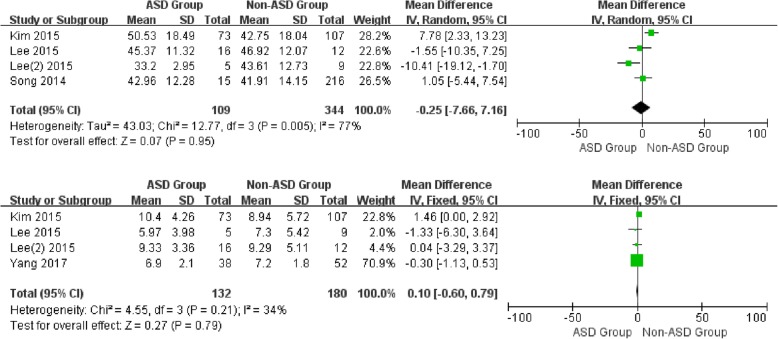
Forest plot of ASD group and non-ASD group: Comparison of preoperative cervical lordosis range of motion (above) and preoperative segment lordosis range of motion (below)

For the preoperative data, there was no significant difference in CL between patients with and without ASD (WMD -2.19, CI -4.87, 0.48, *P* = 0.11) associated with moderate heterogeneity (*I*^2^ = 52%). Similarly, no significant difference was detected in SL (WMD -0.69, CI -1.61,0.22, *P* = 0.14), cervical range of motion (WMD -0.25, CI -7.66, 7.16, *P* = 0.95), and segmental range of motion (WMD 0.10, CI -0.60, 0.79, *P* = 0.79) with different heterogeneity (*I*^2^ = 0%, *I*^2^ = 77%*, I*^2^ = 34%, respectively) (Figs. 2 and 3).

**Fig. 4:**
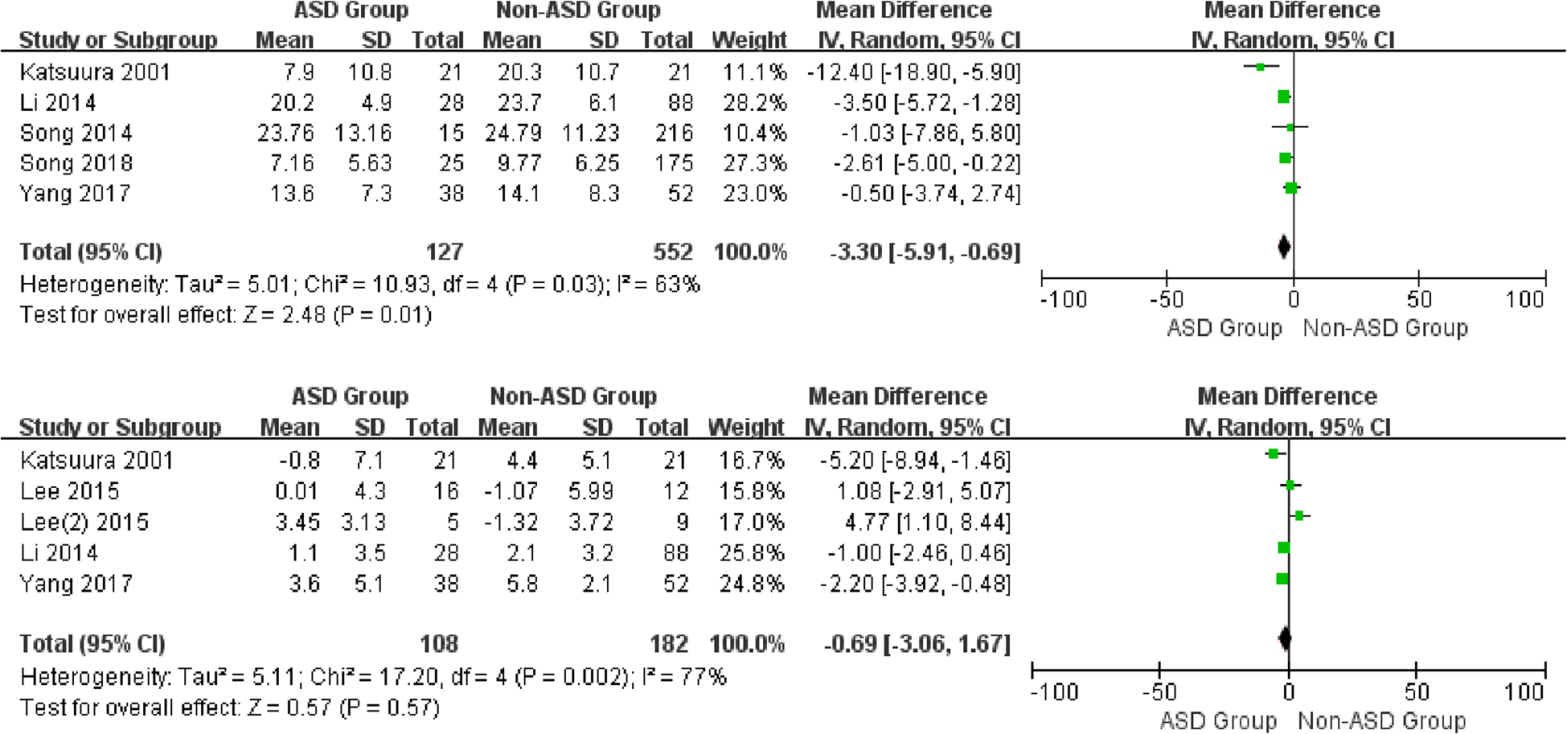
Forest plot of ASD group and non-ASD group: Comparison of postoperative cervical lordosis (above) and postoperative segmental lordosis (below)

**Fig. 5:**
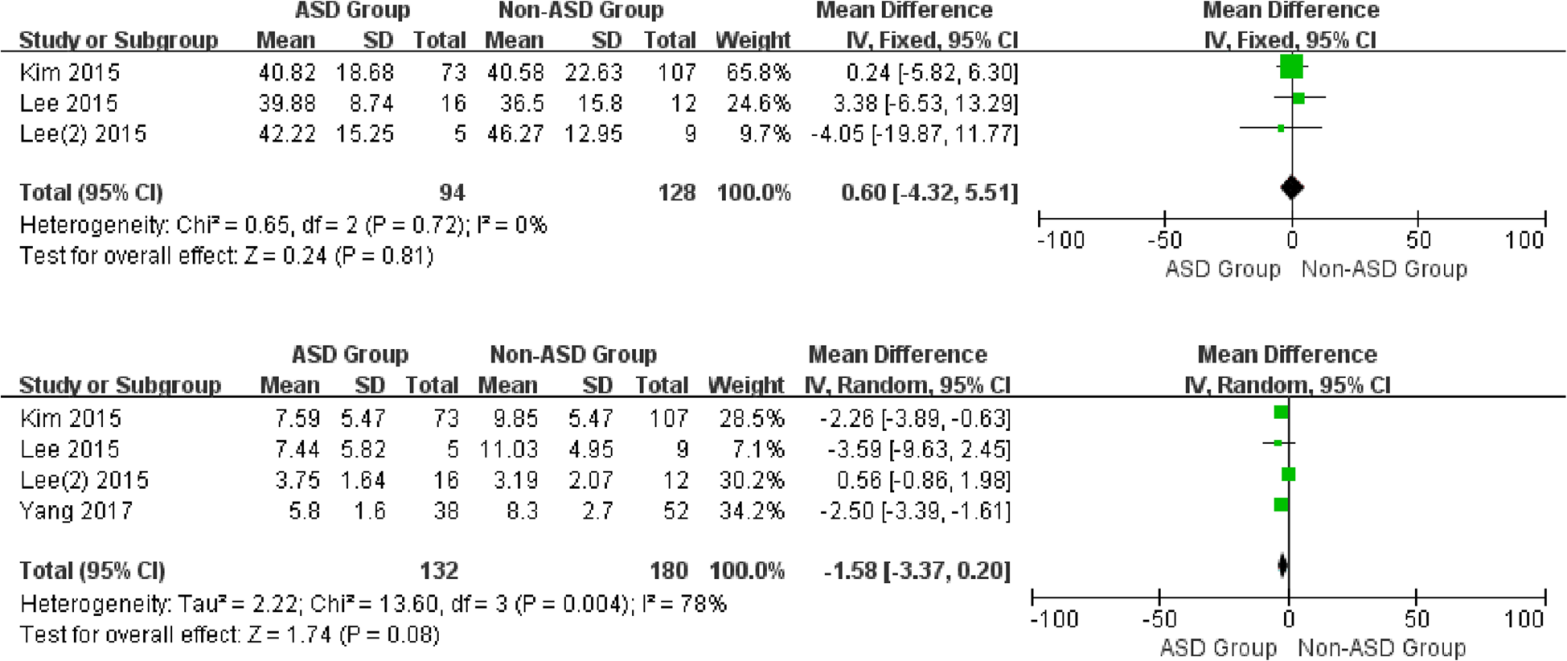
Forest plot of ASD group and non-ASD group: Comparison of postoperative cervical lordosis range of motion (above) and postoperative segmental lordosis range of motion (below)

For the postoperative data, there was no significant difference in SL (WMD -0.69, CI -3.06, 1.67, *P* = 0.57), cervical range of motion (WMD 0.60, CI -4.32, 5.51, *P* = 0.81), and segmental range of motion (WMD -1.58, CI -3.37, 0.20, *P* = 0.08) between the two groups with distinctive heterogeneity (*I*^2^ = 77%, *I*^2^ = 0%*, I*^2^ = 78%, respectively). It is noted that a significant difference was shown in CL between patients with and without ASD (WMD -3.30, CI -5.91, − 0.69, *P* = 0.01) associated with moderate heterogeneity (*I*^2^ = 63%) (Figs. 4 and 5).

### Clinical assessment

**Fig. 6:**
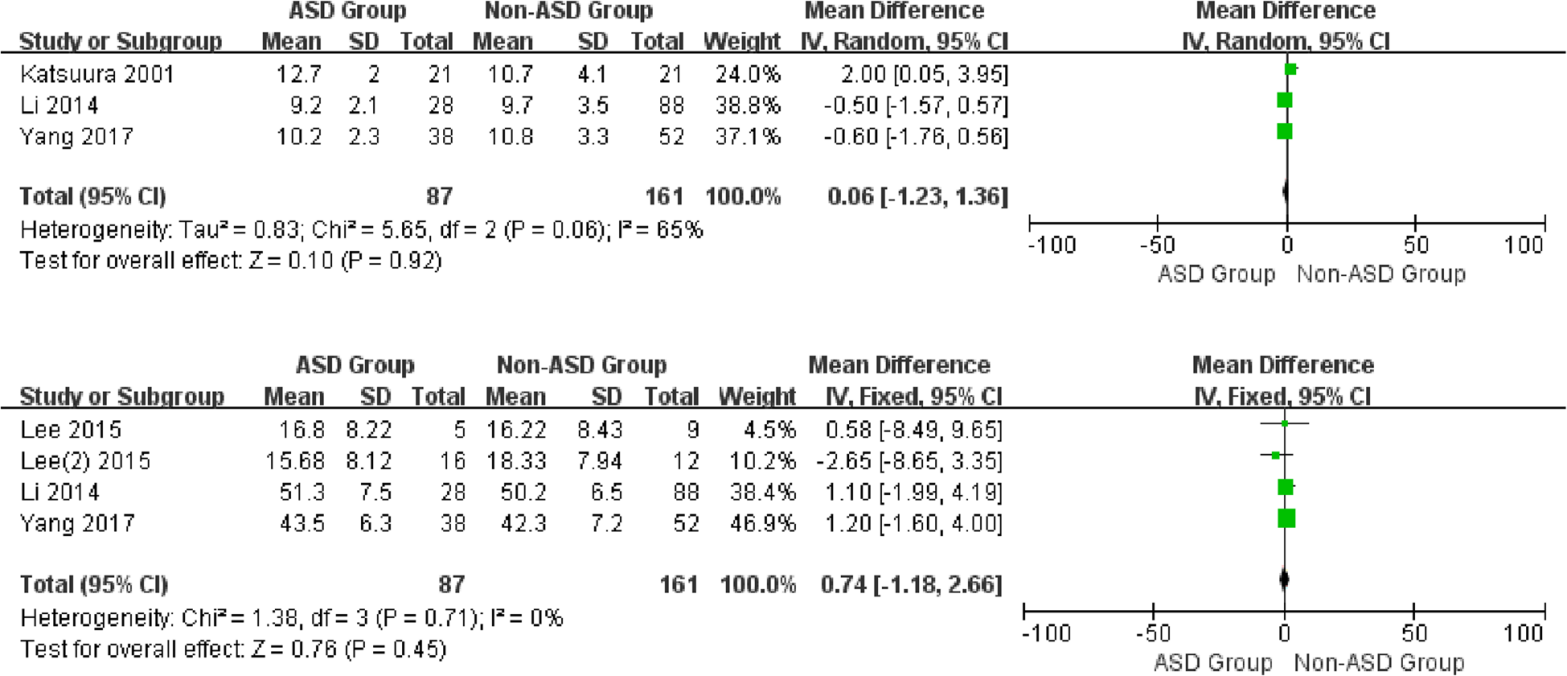
Forest plot of ASD group and non-ASD group: Comparison of preoperative JOA scores (above) and preoperative NDI scores (below)

**Fig. 7:**
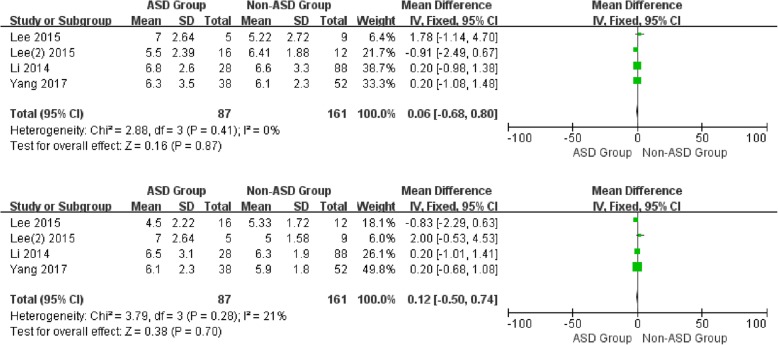
Forest plot of ASD group and non-ASD group: Comparison of preoperative VAS-Neck scores (above) and postoperative VAS-Arm scores (below)

For the preoperative data, there was no significant difference in JOA and NDI between patients with and without ASD (WMD 0.06, CI -1.23, 1.36, *P* = 0.92) (WMD 0.74, CI -1.18, 2.66, *P* = 0.45) associated with moderate and low heterogeneity, respectively (I2 = 65%, I2 = 0%). Similarly, no significant difference was detected in VAS-neck (WMD 0.06, CI -0.68,0.80, *P* = 0.87) and VAS-arm (WMD 0.12, CI -0.50, 0.74, *P* = 0.70) with low heterogeneity (I2 = 0%, I2 = 21%) (Figs. 6 and 7).

**Fig. 8:**
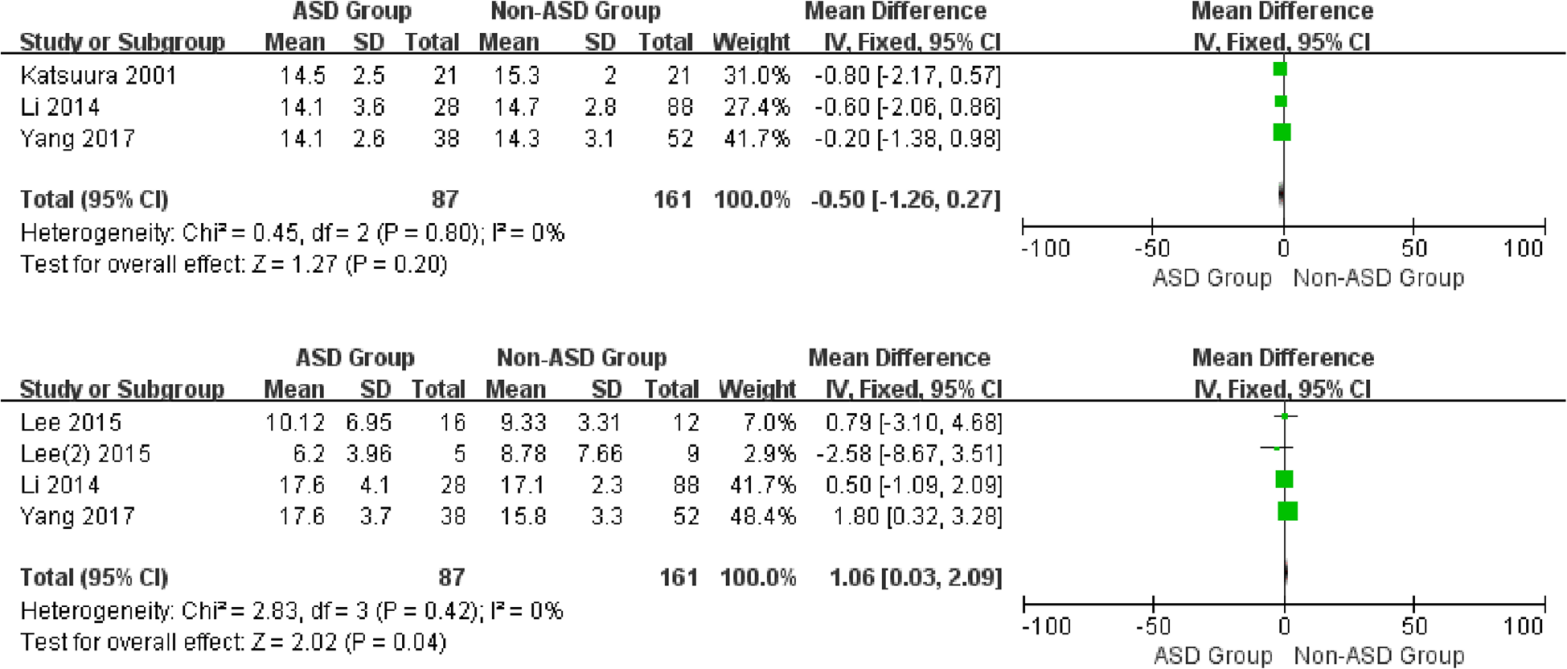
Forest plot of ASD group and non-ASD group: Comparison of postoperative JOA scores (above) and postoperative NDI scores (below)

**Fig. 9:**
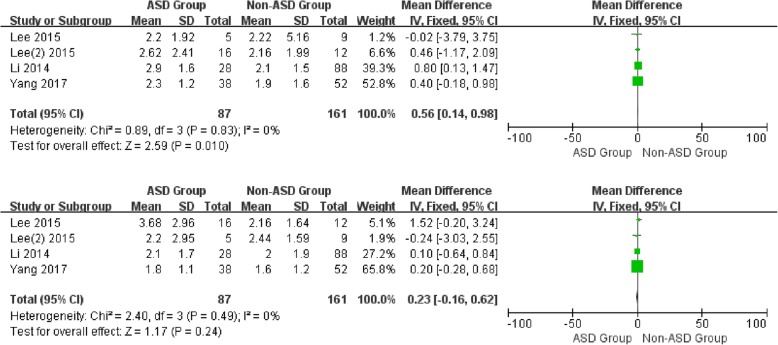
Forest plot of ASD group and non-ASD group: Comparison of postoperative VAS-Neck scores (above) and postoperative VAS-Arm scores (below)

For the postoperative data, there was no significant difference in JOA (WMD -0.50, CI -1.26, 0.27, *P* = 0.20) and VAS-arm (WMD 0.23, CI -0.16, 0.62, *P* = 0.24) between the two groups with low heterogeneity (I2 = 0%, I2 = 0%). It is noted that a significant difference was shown in NDI and VAS-neck between patients with and without ASD (WMD 1.06, CI 0.03, 2.09, *P* = 0.04) (WMD 0.56, CI 0.14, 0.98, *P* = 0.01) associated with low heterogeneity (I2 = 0%, I2 = 0%) (Figs. 8 and 9) (Additional file 1).

## Discussion

Anterior cervical surgery including anterior cervical discectomy and fusion (ACDF), anterior cervical corpectomy and fusion (ACCF), and cervical disc arthroplasty (CDA) have all proved to be effective procedures for the treatment of cervical degenerative disease. Operative treatment can dramatically bring the imbalanced sagittal curve closer to a normal lordotic alignment and increase disc height, relieving neck pain and improving quality of life [23, 24]. However, various complications such as dysphagia, C5 nerve palsy, implant failure, and adjacent segment disease may also occur after surgery [25].

Adjacent segment disease (ASD) is a common complication after cervical surgery and is defined as the development of new clinical symptoms that correspond to the radiographic change to the level of the previous spinal fusion [26]. Patients with postoperative ASD may have uncomfortable symptoms including neck pain, stiffness and activity limitation. In this study, we revealed compared with non-ASD, patients with ASD had prominently worse NDI and VAS-neck scores. The symptomatic ASD after ACDF was reported to be at a relatively constant incidence of 2.9% per year [27], however, our meta-analysis revealed that the integrated incidence of ASD was 24.5% (221/901) from the seven articles included, all of which had at least 2 years follow-up. We concluded that this may be attributed to the definition of ASD, which requires both radiographic and symptomatic ASD. Generally, radiographic alteration of ASD can be detected via MRI after cervical surgery, but only a fraction may develop into symptomatic ASD that requires reoperation. Wu et al. [28] analyzed an 11-year national database in Taiwan and found that while the incidence of the repeated ACDF for ASD was approximately 0.8%, as much as 5.6% patients received a second operation at the end of the 10-year cohort. This indicated that the risk of reoperation for ASD may increase over time.

The pathology of ASD is a complicated process and may be attributed to multiple factors. Nunley et al. [29] reported that osteopenia (OD = 9.35) and documented lumbar degenerative disc disorder (OD = 5.13) were two independent risk factors for ASD in patients undergoing total disc replacement. Yu et al. [30] found that older age, poor restoration of postoperative cervical lordosis, and a plate to disc distance less than 5 mm may result in ASD after cervical fusion. Lee et al. [31] revealed that patients with one- or two- level anterior arthrodesis and those who smoked were more likely to receive an adjacent segment surgery within 10 years. Recently, some studies indicated that postoperative restoration of sagittal balance may be related to clinical outcomes [32]. Furthermore, it was reported that sagittal balance of the cervical spine may be associated with ASD. Alhashash et al. [33] demonstrated that ASD is more likely to occur in single level fusion and that restoration and preservation of cervical sagittal alignment can significantly reduce the risk of ASD. Another study also verified that malalignment of the cervical spine following anterior cervical fusion can lead to the development of ASD [34]. Conversely, Park et al. [35] found that several radiographic parameters were unrelated to postoperative ASD, indicating that ASD is associated with a natural degenerative process instead of operative complications. In our meta-analysis, most sagittal balance parameters showed no significant difference between ASD and non-ASD patients. It is worth noting that a significant difference was detected in postoperative CL between two groups (OR = -3.32, *P* = 0.02), which indicates that CL restoration may be correlated with the development of postoperative ASD.

The natural curvature of the cervical spine maintains a lordotic shape to maintain the wedge-shaped cervical vertebrae and compensate for the kyphotic curvature of the thoracic spine [36]. Nevertheless, increasing age and long-term improper posture may alter the normal alignment of the cervical spine, and even result in progressive cervical kyphosis. Furthermore, the deformity can lead to draping of the spinal cord against the vertebral bodies and increase longitudinal cord tension, which subsequently compresses the spinal cord and nerve root [37]. Although symptoms can be alleviated via the surgical procedures including ACCF and CDA, simple decompression without the correction of deformity, especially cervical kyphosis, may not result in satisfied outcomes. It was considered that insufficient correction of sagittal balance may contribute to the pathology of ASD due to increased anterior loads and pressure [38]. Biomechanical results showed that intradiscal stress and ROM at the adjacent segment changed more in the postoperative model with less lordosis, which illustrated that decreased lordosis may result in alteration of the adjacent segment and subsequently lead to the onset of ASD [39]. Despite several sagittal balance parameters in this meta-analysis, our results showed no significant difference between ASD and non-ASD patients, and the crucial parameter “postoperative CL” differed significantly. It has been suggested that CL is the optimal parameter to depict cervical alignment and that it correlates with several other sagittal balance parameters like T1 slope and SVA [40]. Indeed, C2~7 SVA is regarded as an important sagittal balance parameters, however, because of the limited data the authors cannot analyze the impact of C2~7 SVA on ASD. On the other hand, CL proved to be more important in relation to long-term clinical outcomes [41]. Hence, we speculate that the progression of ASD may result from the abnormal stress distribution of adjacent segments and the increased tension in the posterior column when moving the segments, caused by decreased CL after surgery.

### Limitations

There are some limitations in this meta-analysis – first, only seven articles were enrolled. This is because few studies have focused on the relationship between sagittal balance and ASD and grouped patients by ASD and non-ASD. Second, this study consisted of one prospective cohort study and six retrospective studies, which may lead to less powerful results compared with randomized controlled trials. Third, due the limited enrolled studies, the heterogeneity in some results were substantial, which may lead to unpowered evidence. Forth, though C2~7 SVA was an important sagittal balance parameters, insufficient data from published articles cannot be analyzed in this study. Fifth, the original data, especially the complete sagittal balance parameters from the included articles, failed to be obtained in this study, which may limit the credibility and reliability of our results. Lastly, since ASD measurement is unstandardized across the different countries included, this may cause some discrepancies between the results. Therefore, more randomized controlled trials of high quality that focus on more detailed sagittal balance parameters are needed to analyze the association between sagittal balance and ASD.

## Conclusion

The sagittal balance parameter postoperative CL is a crucial factor which may predict the development of ASD. Sufficient restoration of CL may decrease the incidence of ASD in patients undergoing anterior cervical surgery for degenerative cervical disease. Due to the limited studies and moderate heterogeneity, the conclusions of the present study need to be interpreted with caution. More studies with strong evidence and adequate parameter were needed to verify our conclusion in future.

## Additional file

Additional file 1: Publication bias and sensitivity analysis. (TIF 7608 kb)

## Data Availability

The datasets used and / or analyzed during the current study are available from the corresponding author on reasonable request.

## Abbreviations

ACCF: Anterior cervical corpectomy and fusion
ACDF: Anterior cervical discectomy and fusion,
ASD: Adjacent segment disease
CDA: Cervical disc arthroplasty
TDR: Total disc replacement
WMD: Weighted mean difference
