# Supplementary figures and images for: Association between sagittal balance and adjacent segment degeneration in anterior cervical surgery: a systematic review and meta-analysis
Source: BMC Musculoskelet Disord. 2019 Sep 14;20:430. doi: 10.1186/s12891-019-2800-0 (PMC6745077; doi:10.1186/s12891-019-2800-0)

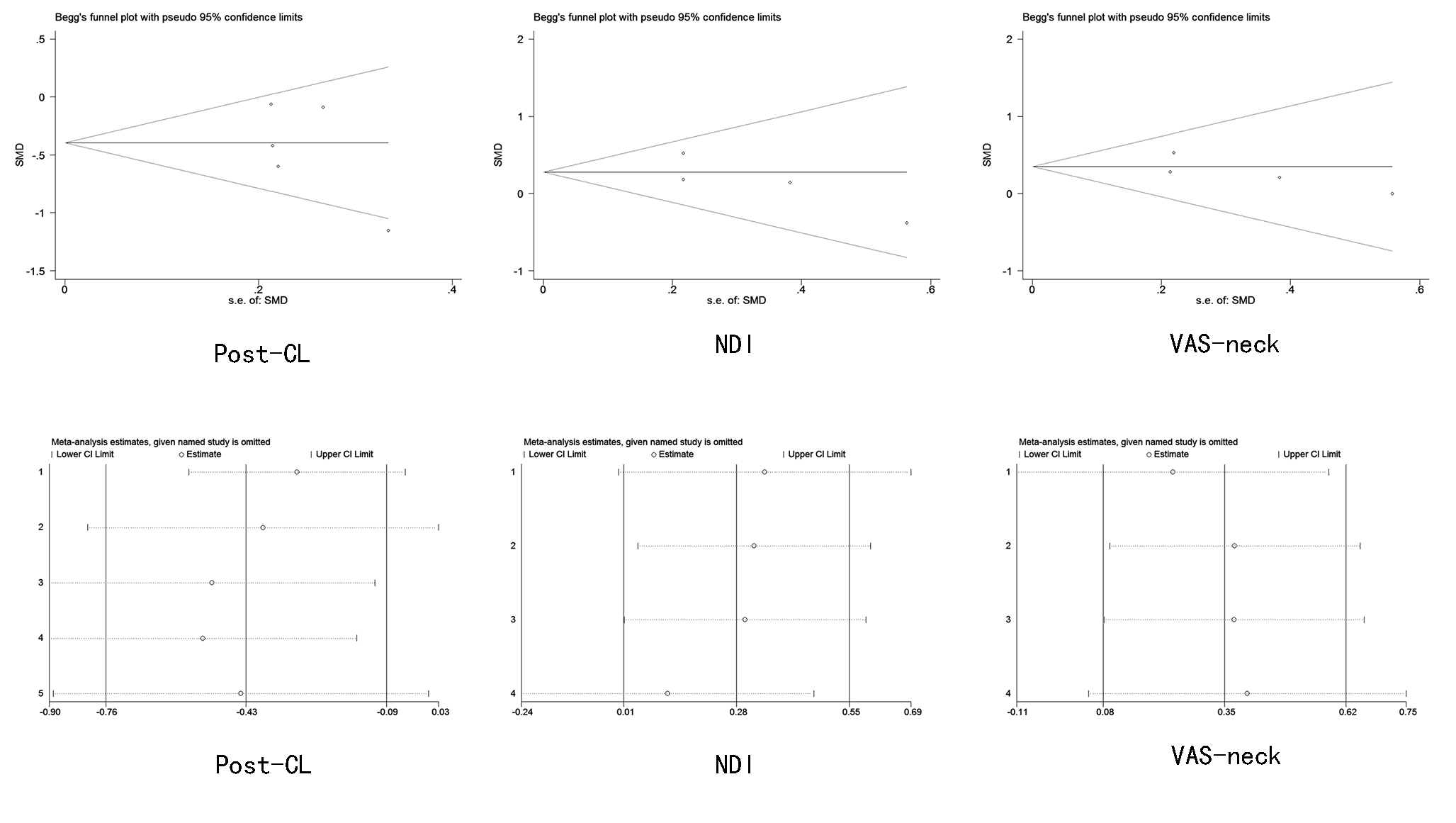

Supplement: Supplementary file 1 — Publication bias and sensitivity analysis. (TIF 7608 kb) [file 12891_2019_2800_MOESM1_ESM.tif]
